# Worth the Wait? Time Course of Supine Shifts in Body Water Compartments on Variables of Bioelectrical Impedance Analysis

**DOI:** 10.2478/joeb-2022-0014

**Published:** 2023-01-08

**Authors:** Jeremy B. Ducharme, Holly Hall, Zachary J. Fennel, Avadney Gerard-Osbourne, Jonathan M. Houck, Chloe Clark, Ann L. Gibson

**Affiliations:** 1 Department of Health, Exercise, and Sports Sciences, University of New Mexico, Albuquerque, NM, USA; 2 Department of Science, Husson University, Bangor, ME, USA

**Keywords:** BIA, TBW, ICW, ECW, body fat, phase angle

## Abstract

Bioelectrical impedance analysis (BIA) reference values are based on supine assessments. Little is known regarding the effects of time course shifts in body water compartments after assuming a supine position. The aim of this study was to characterize these effects and provide recommendations regarding the optimal waiting time to perform BIA. Thirty-eight healthy adults underwent BIA via the RJL Quantum Legacy analyzer immediately upon lying down and every 5 minutes for 15 minutes. Differences in resistance (R), reactance (Xc), intracellular (ICW), extracellular (ECW), total body water (TBW), body fat percentage (%BF), and phase angle (PhA) were assessed. There were small but significant increases in R, Xc, and %BF (all p<0.001), as well as small but significant decreases in ICW, ECW, and TBW (all p<0.001) over 15 minutes. No difference was observed for PhA (p=0.065). Average values changed over 15 minutes by +7.14Ω, +1.36Ω, -0.2L, -0.2L, -0.4L, +0.05° and +0.1% for R, Xc, ICW, ECW, TBW, PhA and %BF, respectively. BIA measurements are affected by shifts in body water compartments after assuming a supine position, but these differences lack clinical significance in healthy adults. Technicians working with healthy adults can perform BIA within 15 minutes after participants assume a supine position.

## Introduction

Bioelectrical impedance analysis (BIA) uses one or more weak electrical current(s) (single frequency: SFBIA; multiple frequency: MFBIA, respectively) to determine the resistance (R) and reactance (Xc) of body tissues. BIA can provide useful measurements that aid medical decisions by evaluating changes in intracellular (ICW) and extracellular (ECW) water compartments that sum to derive total body water (TBW) [1]. Also, BIA is often used to estimate and track changes in body fat percentage (%BF) [2], as well as assess an individual’s intracellular health and muscle quality by measuring phase angle (PhA) [3]. Together, these factors have contributed to the growth of BIA as a practical [2], portable [4], and accessible [5] tool to evaluate various health parameters of an individual. Importantly, accurate assessments of BIA are required to provide meaningful interpretations of measurements obtained by BIA [6].

There are significant postural effects on body water compartments when transitioning from vertical and supine positions due to gravity mediated fluid shifts between the periphery and the trunk resulting in differences in measurements of impedance [1,7, 8, 9, 10, 11]. To minimize these changes, guidelines ranging from 4 minutes to greater than 30 minutes of waiting prior to assessments have been suggested after transitioning from one position to the other [9,12,13]. Previous researchers have demonstrated that measurements of body water compartments in healthy adults using a vertical MFBIA device (Seca mBCA 514/515) are not affected by these fluid shifts as no significant differences were observed for values of ECW or TBW up to 15 minutes after transitioning from a supine to a vertical position [1]. Less is known regarding the effect of the time course shifts in body water compartments after transitioning from a vertical to a supine position [9]. Importantly, reference values for BIA are based on supine assessments of bioelectrical impedance, typically obtained via SFBIA [14, 15, 16]. Therefore, in order to be compared to normative data, individuals undergoing BIA for research or clinical purposes are likely to be assessed in the supine position after transitioning from a vertical position (i.e., walked to the testing station, or following standing measurements of height and weight) [14].

Our lab previously demonstrated significant differences up to 30 minutes after assuming a supine position for both ICW (p <0.001) and ECW (p <0.001) using a supine tetrapolar MFBIA device (Hydra 4200, Xitron Technologies, San Diego, CA, USA) in healthy adults (age: 33±14 yrs., BMI: 24.3±3.9 kg/m^2^) [9]. However, initial measurements immediately after assuming the supine position were not assessed, and the consequences of these differences on other clinical makers of health such as PhA and %BF have yet to be investigated. Researchers have suggested that changes in R and Xc are greatest immediately upon transitioning into the supine position [17,18]. Additionally, new BIA devices such as the RJL Quantum Legacy analyzer (released August 2019 by RJL Systems, Clinton Township, MI) have undergone technological advancements and algorithm updates that may make direct comparisons with previous results using older BIA devices difficult. Therefore, the purpose of this study was to address this gap in the literature by investigating, the effect of supine shifts in body water compartments after transitioning from a vertical to a supine position on measurements of R and Xc, and the corresponding calculations of ICW, ECW, TBW, PhA, and %BF as assessed by the RJL Quantum Legacy analyzer. Our primary aim was to characterize the effect of supine shifts in body water compartments on BIA measurements and provide recommendations for technicians and clinicians alike regarding the optimal waiting time after assuming a supine position to perform BIA.

## Materials and methods

### Participants

Thirty-eight (16 men, 22 women) healthy, young adults participated in the study and their demographics are presented in Table 1. These individuals included college athletes, students, as well as local community members of varying fitness levels. All participants were between the ages of 18 and 45 years old and were screened to ensure that they met inclusion criteria of: no pacemaker or other metal surgical hardware (e.g., cardiac defibrillator, plates, or rods), no amputations or missing appendages, no known heart or kidney disease, no peripheral edema, not undergoing dialysis treatments, and not being prescribed/taking diuretic medication as these are all capable of interfering with BIA testing. Additionally, urine pregnancy tests were given to all female participants (n=22) as pregnancy was an exclusion criterion. All participants provided written informed consent before enrolling in the study, and all procedures were approved by the university’s Institutional Review Board for human subject research.

**Table 1:** Participant demographics (mean ± SD).

	Men (n=16)	Women (n=22)	Total (N=38)
**Age (years)**	24.2 ± 3.9	22.8 ± 4.3	23.4 ±4.1
**Height (cm)**	177.7 ± 4.7	167.7 ± 9.0	171.9 ± 8.9
**Body Mass (kg)**	78.9 ± 10.2	65.0 ± 12.1	70.9 ± 13.2
**Body Mass Index (kg/m^2^)**	25.0 ± 4.6	23.1 ± 1.5	23.8 ± 3.3

### Experimental Design

Using a cross-sectional study design, eligible participants were instructed to arrive to the university’s Exercise Physiology Laboratory for a single session of data collection in a euhydrated state, urine specific gravity ≤1.020 [19] as assessed by a refractometer (Model A300, ATAGO Co., Tokyo, Japan). Prior to their appointment, each participant was reminded to following pre-test guidelines consisting of

1. voiding completely within 30 min of being tested,
2. refraining from eating or drinking within 4 hours of testing,
3. avoiding strenuous exercise within 12 hours of testing,
4. refraining from consuming alcohol within 48 hours of testing.

Participants removed all jewelry prior to testing. After participants had voided their bladder and bowels, measurements of height and body mass were obtained without shoes using a stadiometer and scale (Seca GMBH & Co. Hamburg, Germany), respectively. To minimize possible effects of diurnal variation on body water compartments, all assessments took place in the morning between 06:00 and 10:00.

### Bioelectrical Impedance Analysis

The RJL Quantum Legacy was used to measure participants’ R, Xc and PhA at 50 kHz using a tetrapolar wrist-to-ankle electrode configuration. Measurements were taken immediately upon assuming a supine position on a soft, non-conductive foam mat and every 5 minutes for 15 minutes so that a total of four time points (0, 5, 10, and 15 minutes) had been recorded. Participants remained motionless throughout the duration of the test with their arms and legs abducted enough to minimize contact between the torso and thighs, respectively. Standard skin preparation was performed before electrode placement for tetrapolar wrist-to-ankle electrode configuration assessments. All measurements with the analyzer were taken on the participant’s right side. Estimations of ECW, ICW, TBW, and %BF were determined by entering participant’s R, Xc, age, sex, ethnicity, and body mass into the RJL’s BC 4 proprietary software. The same analyzer was used throughout the duration of the study and successfully passed calibration testing prior to measuring R, Xc, and PhA for each participant per manufacturer recommendations.

The NIH recommends resting supine for 10 minutes prior to BIA assessments [11]; therefore, two additional measurements, making for a total of three measurements, were taken at 10 minutes to determine the reliability and accuracy of the RJL Quantum Legacy analyzer to measure R, Xc, and PhA. Alligator clips were removed then reattached to their respective electrodes prior to each additional measurement taken at 10 minutes.

### Statistical Analyses

To determine sample size, an a priori power analysis was conducted using G*Power software (version 3.1.9.6) [20], using a conservative estimate of effect size (Partial η^2^ =0.045) from a prior study examining the effect of body water compartment shifts in the supine position on TBW [9]. It was estimated that when using a repeated measures ANOVA with one group, four time points, an α-level of 0.05, and a power of 0.80 (1 – β), that 31 participants would be required to detect statistically significant differences in body water compartments after transitioning from a vertical to supine position. To account for the possibility of attrition, inability to screen into the study, or unusable data, 38 participants were recruited and participated in the study.

Data are reported as mean ± standard deviation (SD). Separate one-way repeated measures ANOVAs were used to evaluate if there were differences in R, Xc, PhA, ECW, ICW, TBW, and %BF throughout 15 minutes of supine rest after transitioning from a vertical to a supine position. Generalized η^2^ (η^2^_G_) was reported to quantify the observed effect size for the repeated measures ANOVA comparisons, where small=0.01; medium=0.06; and large=0.14 [21, 22, 23]. The Greenhouse–Geisser correction was used whenever the Mauchly’s Test of Sphericity was violated. Three post-hoc two-tailed paired sample t-tests with Bonferroni corrections for multiple comparisons (0 vs 5 minutes, 5 vs 10 minutes, and 10 vs 15 minutes) were used to determine if there were mean differences between time points for each variable of interest (R, Xc, ICW, ECW, TBW, PhA, and %BF). Individual differences and systematic biases between time points for each variable of interest were evaluated using Bland-Altman 95% limits-of-agreement (LoA) plots [24] with a line of best fit superimposed within each plot [25], respectively. The magnitude of variation amongst variables of interest that was explained by their respective average was determined using the coefficient of determination (R^2^). An α of 0.05 was used as a threshold for statistical significance. Using the three measurements at 10 minutes in the supine position, Cronbach’s alpha reliability coefficient and standard error of estimates were calculated to evaluate the reliability and accuracy of the RJL Quantum Legacy analyzer, respectively.

Analyses were completed using RStudio version 2022.02.2+485 "Prairie Trillium" for Windows. Case diagnostics were performed to ensure assumptions of sphericity and normality were met. Potential influential outliers were examined by comparing the Cook’s distance values. All graphical representations of data were produced in Prism (version 6; GraphPad Software Inc., La Jolla, CA).

### Informed Consent

Informed consent has been obtained from all individuals included in this study.

### Ethical Approval

The research related to human use has been complied with all relevant national regulations, institutional policies and in accordance with the tenets of the Helsinki Declaration, and has been approved by the authors’ institutional review board or equivalent committee.

## Results

Data met the assumption of normality after examining the standardized residuals. Mauchly’s Test of Sphericity indicated that the assumption of sphericity had been violated; consequently, Greenhouse–Geisser corrections were used. Case diagnostics indicated no influential outliers in the data set. All participants met the hydration status requirement. The majority (52.6%; n=20) of the participants in this study were White, 26.3% (n=10) were Hispanic, 13.2% (n=5) were Black, and 7.9% (n=3) were Native American. Cronbach’s alpha reliability coefficient showed that the RJL Quantum Legacy analyzer had excellent reliability across the three assessments measured after 10 minutes of rest in the supine position for R (0.999), Xc (.998), PhA (0.998). The standard error of estimate for the RJL Quantum Legacy analyzer was 1.46Ω for R, 0.37Ω for Xc, and 0.04° for PhA.

**Fig. 1:**
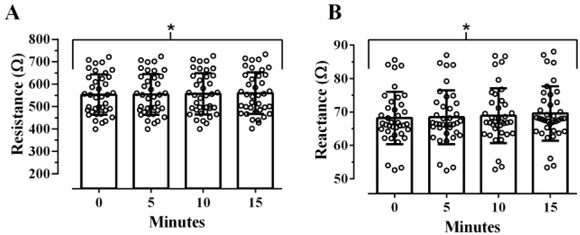
Effect of time since transitioning from a vertical to a supine position on resistance (A), and reactance (B) over 15 minutes as assessed via the RJL Quantum Legacy analyzer (N=38). Open circles represent individual responses. *Denotes a statistically significant increase from baseline over the course of 15-minutes resting in a supine position (p<0.05).

Figure 1A shows that there was a significant difference albeit small effect of time on the assessment of R, F(1.76, 65.16)=74.091, p<0.001, η^2^_G_<0.001. Post-hoc comparisons showed no significant difference between baseline and 5 minutes (p=0.228), but R significantly increased from 5 to 10 minutes (p<0.001), and from 10 to 15 minutes of resting supine (p<0.001) (Table 2). Average R measured immediately after transitioning from a vertical to supine position was 7.14 Ω less compared to R measured after 15 minutes of resting in the supine position. Similarly, there was a significant difference and small effect of time on the assessment of Xc, F(1.24, 46.01)=20.958, p<0.001, η^2^_G_=0.004 (Fig. 1B). Again, post-hoc comparisons showed no significant difference between baseline and 5 minutes (p=0.541), but Xc significantly increased from 5 to 10 minutes (p=0.009) and from 10 to 15 minutes of resting supine (p<0.001) (Table 2). Average Xc measured immediately after transitioning from a vertical to supine position was 1.36Ω less than Xc measured after 15 minutes of resting in the supine position.

**Table 2:** Average bioelectrical variables as assessed by the RJL Quantum Legacy device (N=38)

Variable	Time since transitioning from a vertical to a supine position			
	0 min	5 min	10 min	15 min
**R (Ω)**	553.2±90	554.1±91	556.9±92	560.4±93*
**Xc (Ω)**	68.2±7.8	68.4±8.1	68.9±8.1*	69.6±8.1*
**ICW (L)**	20.9±4.8	20.9±4.8	20.8±4.8*	20.7±4.8*
**ECW (L)**	16.5±2.9	16.5±2.9	16.4±2.9*	16.3±2.9*
**TBW (L)**	37.4±7.5	37.4±7.6	37.2±7.6*	37.0±7.6*
**PhA (°)**	7.13±0.9	7.14±0.9	7.16±0.9	7.18±0.9
**BF (%)**	22.4±6.8	22.4±6.8	22.5±6.8*	22.5±6.8*

R = resistance, Xc = reactance, ICW = intracellular water, ECW = extracellular water, TBW = total body water, PhA = phase angle, BF = body fat. *Denotes a statistically significant difference compared to the previous time point (p<0.05).

**Fig. 2:**
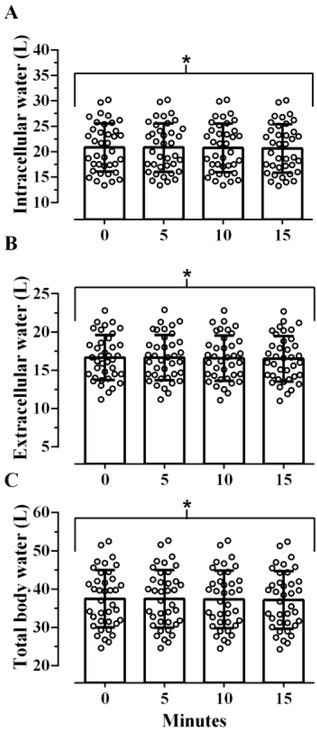
Effect of time since transitioning from a vertical to a supine position on intracellular water (A), extracellular water (B), and total body water (C) over 15 minutes as assessed via the RJL Quantum Legacy analyzer (N=38). Open circles represent individual responses. *Denotes a statistically significant decrease from baseline over the course of 15-minutes resting in a supine position (p<0.05).

Figure 2A shows that there was a significant difference but small effect of time on the estimation of ICW, F(2.37, 87.78)=58.580, p<0.001, η^2^_G_<0.001. Post-hoc comparisons showed no significant difference between baseline and 5 minutes (p>0.999), but ICW significantly decreased from 5 to 10 minutes, (p<0.001), and from 10 to 15 minutes of resting supine (p<0.001) (Table 2). Average ICW measured immediately after transitioning from a vertical to supine position was 0.2L greater than ICW measured 15 minutes later. There was also a significant difference but small effect of time on the estimation of ECW, F(1.62, 59.83)=61.093, p<0.001, η^2^_G_<0.001 (Fig. 2B). Similarly, post-hoc comparisons showed no significant difference between baseline and 5 minutes (p>0.999), but ECW significantly decreased from 5 to 10 minutes, (p<0.001,), and from 10 to 15 minutes of resting supine (p<0.001) (Table 2). The difference between the average ECW measured immediately after transitioning from a vertical to supine position was 0.2 L greater than ECW measured 15 minutes later. Like the effect of time on ICW and ECW, there was a significant difference albeit small effect of time on the estimation of TBW, F(1.93, 71.43)=68.346, p<0.001, η^2^_G_<0.001 (Fig. 2C). Like the previous responses, post-hoc comparisons showed no significant difference between baseline and 5 minutes (p>0.999), but TBW significantly decreased from 5 to 10 minutes, (p<0.001), and from 10 to 15 minutes of resting supine (p<0.001) (Table 2). Average TBW measured immediately after transitioning from a vertical to supine position was 0.4 L greater than TBW measured after 15 minutes of resting in the supine position.

**Fig. 3:**
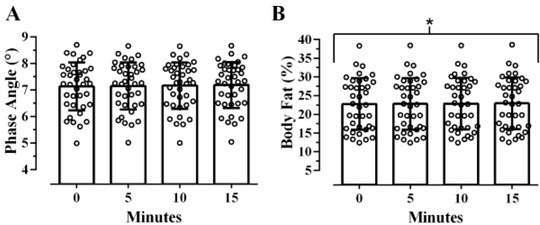
Effect of time since transitioning from a vertical to a supine position on phase angle (A), and body fat percentage (B) over 15 minutes as assessed via the RJL Quantum Legacy analyzer (N=38). Open circles represent individual responses. *Denotes a statistically significant increase from baseline over the course of 15-minutes resting in a supine position (p<0.05).

Similar to the previous variables of interest, a small effect was observed when assessing the differences between the various times points used to measure PhA in the present study, but unlike the previous responses, no statistical differences were observed, F(1.2, 44.51)=3.401, p=0.065, η^2^_G_<0.001 (Fig. 3A). The difference between the average PhA measured immediately after transitioning from a vertical to supine position was 0.05° less than PhA measured after 15 minutes of resting in the supine position.

A significant difference albeit small effect was observed for time on the estimation of %BF, F(1.52, 56.41)=37.727, p<0.001, η^2^_G_<0.001 (Fig. 3B). Post-hoc comparisons showed no significant difference between baseline and 5 minutes (p>0.999), but %BF significantly increased from 5 to 10 minutes, (p<0.001), and from 10 to 15 minutes of resting supine (p<0.001) (Table 2). The difference between %BF estimated immediately after assuming the supine position and estimated 15 minutes later was 0.1%.

Bland–Altman plots were used to evaluate the individual differences between time points for each variable of interest.

**Fig. 4:**
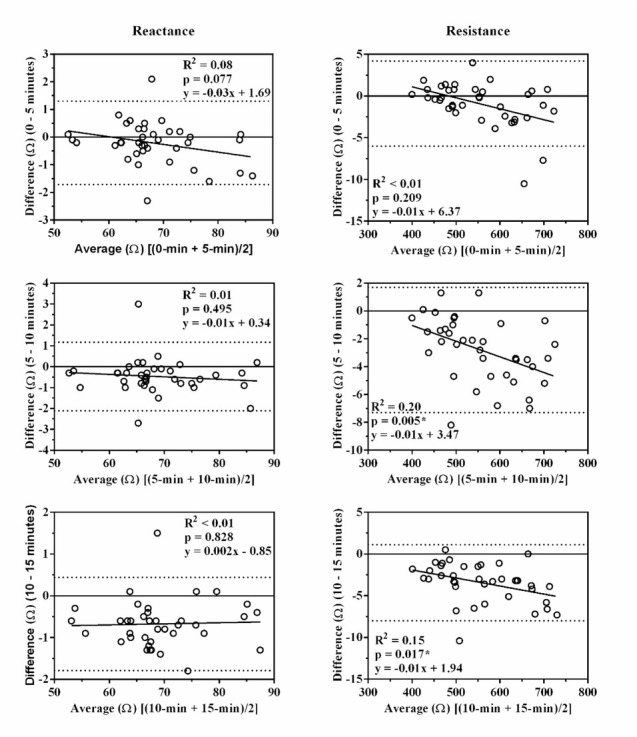
Bland and Altman plots of individual differences for the effect of time since transitioning from a vertical to a supine position on reactance (left side) and resistance (right side) (N = 38). The solid line represents a mean difference (bias) of zero. The dashed lines denote the upper and lower limits-of-agreement (bias ± [1.96×SD of the bias]). R^2^ represents the amount of variance explained by the average resistance or reactance. *p<0.05 significant systematic bias.

**Fig. 5:**
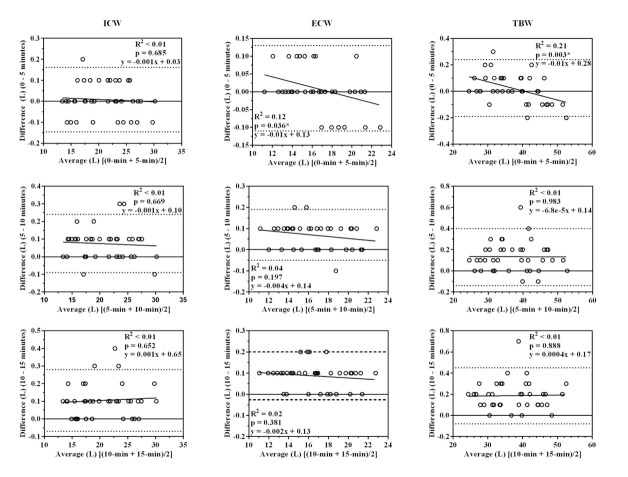
Bland and Altman plots of individual differences for the effect of time since transitioning from a vertical to a supine position on intracellular water (ICW: left side), extracellular water (ECW: middle), and total body water (TBW: right side) (N = 38). The solid line represents a mean difference (bias) of zero. The dashed lines denote the upper and lower limits-of-agreement (bias ± [1.96×SD of the bias]). R^2^ represents the amount of variance explained by the average ICW, ECW, or TBW. *p<0.05 significant systematic bias.

**Fig. 6:**
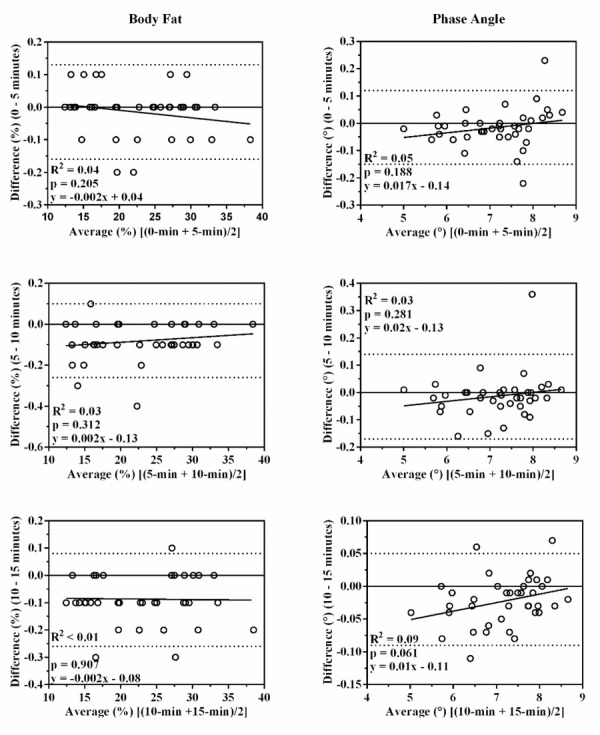
Bland and Altman plots of individual differences for the effect of time since transitioning from a vertical to a supine position on body fat (left side) and phase angle (right side) (N = 38). The solid line represents a mean difference (bias) of zero. The dashed lines denote the upper and lower limits-of-agreement (bias ± [1.96×SD of the bias]). R^2^ represents the amount of variance explained by the average value. *p<0.05 significant systematic bias.

Similar to the average group comparisons, the magnitudes of the LoA between time points for each variable were relatively small (Supplementary Material; Figures 4-6) and likely lack clinical significance in healthy adults. In total, 95% of individual differences between time points for each variable fell within the LoA, indicating that differences were normally distributed. Simple linear regression analyses of the Bland–Altman data indicated a systematic bias was observed between 5-10 minutes and 10-15 minutes of resting in the supine position for resistance, so that larger decreases over time occurred for those with larger resistance values for all but 3 and 2 participants, respectively (Supplementary Material; Fig. 4). A systematic bias was also observed for ECW (Supplementary Material; Fig. 5) and TBW (Supplementary Material; Fig. 5) to decrease between 0 and 5 minutes of supine rest for individuals with an ECW and TBW greater than or equal to approximately 17 L, and 36 L, respectively. The clinical importance of these biases is unknown as the LoA were relatively small, indicating that differences between time points may lack clinical significance. No other biases were observed for all other time points and variables (Supplementary Material; Figures 4-6).

## Discussion

The purpose of this study was to investigate the effect of supine shifts in body water compartments after transitioning from a vertical to a supine position on measurements of R, Xc, PhA and estimations of ICW, ECW, TBW, and %BF as assessed by the RJL Quantum Legacy analyzer. The key findings from this study were as follows:

- there is a statistically significant increase in both R and Xc (Fig. 1),
- this corresponds with statistically significant decreases in ICW, ECW, and TBW (Fig. 2), as well as increases in %BF, but no change in PhA (Fig. 3), and
- although statistically significant effects were observed for all variables of interest, excluding PhA, these effects were small (η^2^_G_ <0.01) and lack practical significance.

Specifically, we observed differences between immediate measurements of R and Xc compared with those taken 15 minutes later of +7.14 Ω and +1.36 Ω, respectively, which corresponded to differences of only -0.2 L, -0.2 L, -0.4 L, +0.05° and +0.1% for ICW, ECW, TBW, PhA and %BF, respectively.

Importantly, since BIA is applied at the individual level, the effect of shifts in body water compartments over time on BIA measurements depends on the individual variability as assessed by the agreement between time points at the individual level. There were relatively small LoA between time points for all variables of interest, thus supporting that the average group effect observed in this study is consistent at the individual level (Supplementary Material; Figures 4-6). Together, these findings highlight differences in measurements taken by the RJL Quantum Legacy induced by the time course of supine body fluid stabilization are likely lacking clinical significance in healthy adults. Technicians working with this population can avoid waiting the recommended 4 to 30 minutes [9,12,13] before obtaining measurements of SFBIA after participants transition from a vertical to a supine position when using the RJL Quantum Legacy analyzer.

In agreement with the present study, our lab previously demonstrated statistically significant changes in ICW and ECW, but not TBW, over the course of 30 minutes resting in a supine position [9]. Conversely, the current study observed no difference in ICW or ECW between immediate and after 5 minutes of supine rest (Table 2). Therefore, although Gibson et al. [9] did not measure ICW or ECW immediately upon assuming a supine position, there were differences between the 5- and 15-minute time points of ~0.2 L, similar to responses observed in the current study (Table 2). Importantly, both studies used healthy adults and a difference in ICW and ECW between time points of ~0.2 L is likely of little practical significance for this population. More recently, Thurlow et al. [1] demonstrated with healthy adults that time to equilibrate body water compartments after transitioning from a supine to a vertical position does not affect measures of ECW or TBW when assessed on a stand-on MFBIA device (Seca mBCA 514/515).

The current study builds upon these previous findings by extending them to a SFBIA device (RJL Quantum Legacy) used in the supine position and suggest that for practical purposes, assessments of body water compartments are unaffected when assessed within 15 minutes of assuming a supine position with this analyzer. Of note, the current study, as well as previous reports, only used healthy adults. Therefore, future researchers should investigate the time course of supine body water compartment shifts in a clinical sample as small differences in ICW and ECW may be of practical significance in clinical populations.

While assessments of body water compartments via BIA are frequently used in clinical practice and can aid medical decisions [1], other physiologic variables such as PhA and %BF are often key measurements of BIA [2]. This study provides novel findings that build upon previous literature by demonstrating statistically but not practically meaningful shifts in body water compartments and corresponding clinical markers such as PhA and %BF. Of note, no statistical differences were observed for the assessment of PhA between time points (Fig. 3B; Table 2). Therefore, while PhA = arctan(Xc/R)*(180/π), the statistical differences observed for R and Xc did not affect the calculation of PhA, further emphasizing the small effect of time spent in the supine position on BIA. This finding supports that PhA can be accurately assessed immediately upon assuming a supine position without allowing time for body water compartment stabilization via the RJL Quantum Legacy analyzer. We did observe a statistically significant difference yet small effect (p <0.001, η^2^_G_ <0.001) of time on estimations of %BF, but this equated to only a +0.1% difference between measurements taken immediately after assuming a supine position and those taken 15 minutes later. As previously indicated, a difference of 0.1% is of little practical significance for healthy adults. Therefore, this study supports the use of the RJL Quantum Legacy analyzer immediately upon assuming the supine position for estimations of %BF for healthy adults.

In conclusion, although statistically significant, the trivial differences between time points observed in this study indicates that resting in the supine position for 15 minutes after transitioning from a vertical position prior to SFBIA with the RJL Quantum Legacy analyzer lacks practical significance and is likely unnecessary for healthy adults. Assuming that participants adhere to standard pretest guidelines for BIA and the device has been calibrated in accordance with the manufacturer’s guidelines, it appears that the RJL Quantum Legacy analyzer can be used immediately upon assuming the supine position. There is a growing clinical application of SFBIA, and it is unknown if the findings observed in the present study can be generalized to clinical populations; therefore, more research is warranted to characterize the effect of supine shifts in body water compartments in a clinical sample with the RJL Quantum Legacy analyzer.
